# Sims and Vulnerability: On the Ethics of Creating Emulated Minds

**DOI:** 10.1007/s11948-022-00416-y

**Published:** 2022-11-25

**Authors:** Bartlomiej Chomanski

**Affiliations:** Department of Philosophy, Adam Mickiewicz University, Poznan, Poland

**Keywords:** Whole brain emulation, AI ethics, Moral status of artificial beings, Artificial consciousness, Vulnerability

## Abstract

It might become possible to build artificial minds with the capacity for experience. This raises a plethora of ethical issues, explored, among others, in the context of whole brain emulations (WBE). In this paper, I will take up the problem of vulnerability – given, for various reasons, less attention in the literature – that the conscious emulations will likely exhibit. Specifically, I will examine the role that vulnerability plays in generating ethical issues that may arise when dealing with WBEs. I will argue that concerns about vulnerability are more matters of institutional design than individual ethics, both when it comes to creating humanlike brain emulations, and when animal-like emulations are concerned. Consequently, the article contains reflection on some institutional measures that can be taken to protect the sims' interests. It concludes that an institutional framework more likely to succeed in this task is competitive and poly-centric, rather than monopolistic and centralized.

## Introduction

It might become possible to build artificial minds with the capacity for experience. This raises a plethora of ethical issues, explored, among others, in the context of whole brain emulations (WBE). In this paper, I will take up the problem of vulnerability—given, for various reasons, less attention in the literature—that the emulations will likely exhibit. Specifically, I will examine the role that vulnerability plays in generating ethical issues that may arise when dealing with emulations, and gesture at potential solutions to these issues.

The rest of the paper is divided into the following sections. In Sect. “Whole brain emulation”, I offer some background on the theory behind WBE and some controversies about it. Section “Vulnerability and suffering” discusses ways in which sims created through WBE are vulnerable and the ethical issues this raises. Section “Vulnerability for human like sims” looks at potential solutions to the vulnerability of humanlike sims, Sect. “Vulnerability, institutions, and animal sims” does the same for (nonhuman) animal-like sims. Section “Conclusion” concludes.

## Whole Brain Emulation

Anders Sandberg (2014), who pioneered ethical reflection on this topic, is also among the main proponents of WBE as a feasible route to artificial intelligence (the claim that WBE is not only conceptually possible, but also resting on plausible philosophical foundations, and doable in practice given the technologies we may expect to emerge in a relatively short time-frame). Sandberg explains the project as follows:

The basic idea is to take a particular brain, scan its structure in detail at some resolution, construct a software model of the physiology that is so faithful to the original that, when run on appropriate hardware, it will have an internal causal structure that is essentially the same as the original brain. All relevant functions on some level of description are present, and higher-level functions supervene from these. (p. 439)

An attractive feature of WBE as a scientific enterprise is that it does not require the replication of *every* part of the brain in order to recreate the mind; *structure* (at some level of abstraction) *should suffice.*

Whether this is on the right track is a matter of controversy, as Sandberg, of course, recognizes: “[o]bviously, the eventual feasibility [of WBE] depends on a number of philosophical issues (physicalism, functionalism, non-organicism) and empirical facts (computability, scale separation, detectability, scanning and simulation tractability) that cannot be predicted beforehand; WBE can be viewed as a program trying to test them empirically” (440). The upshot seems to be, however, that WBE is given a sheen of plausibility in virtue of the plausibility of the theories on which it rests.

A similar argument has been made by Tyler Bancroft (2013), who raises the following point in WBE’s favor:

Consciousness is generally accepted by neuroscientists to be a property or product of the brain. Information-processing in the brain is carried out by the combined activity of brain cells (both neurons and glia). Brain cells are physical systems (i.e., the operation of neurons obey the laws of physics), and as such, can be represented mathematically at an arbitrary level of mathematical precision … . These mathematical representations can be solved computationally. As such, the activity of brains can be computationally simulated to an arbitrary level of precision, and we must therefore consider the possibility of consciousness in computational simulations of brains. (p. 417)

In a word, if some popular theories in philosophy of mind and neuroscience turn out to be true, then WBE is feasible.

The thesis is not without criticism. Eric Mandelbaum (2022) has recently provided a useful survey of reasons for endorsing the feasibility of WBE and marshaled powerful arguments against it. Mandelbaum argues, for instance, that there are serious problems with the underlying philosophical theories mentioned by Sandberg above, such as functionalism or “non-organicism.” If consciousness is not a matter of functional relations (e.g. if instead it turned out “that the coding and interchange of information between electrical and chemical formats gives rise to consciousness, and that the specific neural hardware we use is essential to phenomenal consciousness” (Mandelbaum, 2022, p. 9)), then WBE is in trouble, as the level of replication of the brain’s properties that needs to be achieved for a chance of consciousness emerging in the emulated system would turn out to be much more fine-grained (say at the sub-neuronal level) than what proponents of WBE suppose.

This is not the place to adjudicate between these perspectives. I will assume, however, that WBE’s chances of success are high enough that its consequences merit serious ethical reflection. Moreover, other attempts to create consciousness “in the lab,” such as via brain organoids, are also underway, engender similar issues, and may escape theoretical criticisms raised by Mandelbaum.

## Vulnerability and Suffering

There already exists philosophical opposition to building artificial, conscious minds.^1^ Sander Beckers (2018), Thomas Metzinger (2013), and John Basl (2013) have offered a variety of arguments why the project of building artificial consciousness (regardless of the method whereby it’s achieved) is fraught with ethical challenges—connected primarily with the suspicion that, for all we know, it’s possible that in building conscious artificial minds, researchers will inadvertently create artificial suffering on an enormous scale. This might be due to engineering errors as well as the inability to tell when a created being in fact becomes conscious and what sort of consciousness it possesses. Furthermore, given these shortcomings, it is then likely that the researchers will remain ignorant of, and thus unable to stop such suffering—regardless of how enormous it is. This risk of creating unmitigated suffering (potentially of an unprecedented magnitude) is a serious moral problem.

The solutions offered to meet these challenges vary in their stringency, from an outright ban on artificial consciousness research (Metzinger) to more modest mitigation strategies (Basl). Since these arguments apply to any method of creating artificial consciousness, they *a fortiori* can be raised against WBE.

Let us suppose, however, that there are ways of properly limiting artificial suffering; that is, ways of ensuring that brain emulations would not suffer gratuitously, nor will their suffering be unknowable to us. Even so, it is still possible to worry about the ethics of creating sims.

This is because such sims (at least on Sandberg’s account) seem to exist entirely at the mercy of their creators, in a manner not dissimilar from how, e.g., very small children depend for their very survival on others. They can be more easily destroyed, and their whole lives can be upended much more easily by other people’s decisions. Indeed, the sims’ entire world is, to an extent, dependent on the whims of another. That makes them extremely vulnerable.

What, exactly, is vulnerability? It will come as no surprise that philosophers disagree—or at least, offer a number of alternative conceptions of this notion. For the purposes of this paper, I will begin with the definition provided by Nicholas Vrousalis (2013): B is vulnerable to A iff:

(i) B lacks some desideratum x that is a requirement for, or a constitutive feature of, B's flourishing (in which case x is the object of B's need), (ii) B can only obtain x from A, and (iii) A has it within his discretion to withhold x from B. (p. 134)

On this reading, vulnerability sounds like an all-or-nothing concept. But we can transform it into a gradable notion. Let “extreme vulnerability” be Vrousalis’ original definition. Each of his conditions can be modified, to make vulnerability less extreme: e.g., if we increase the number of agents who can provide B with x (C, D, …, Z), then assuming they are independent^2^ from one another, the bigger the number of such agents, the less vulnerable B is. If we decrease the importance of x (e.g. from being constitutive of B’s flourishing to being very helpful towards achieving it)—the less important x is, the less vulnerable B is. Finally, A’s discretion could also be more or less complete, from entirely arbitrary to being subject to various conditions. The less arbitrary A’s exercise of this discretion, the less vulnerable (to A) B is.

On this conception, sims qualify as especially vulnerable, relative to other members of society.^3^ This is because, as Sandberg puts it:

the software and data constituting [the sims] and their mental states can be erased or changed by anybody with access to the system on which they are running. Their bodies are not self-contained and their survival is dependent upon hardware they might not have causal control over. They can also be subjected to undetectable violations such as illicit copying. (Sandberg, 2014, p. 452)

A similar line of thought is also captured by Eric Schwitzgebel & Mara Garza (2015), who argue that the creators of conscious sims whose lives are entirely virtual have “godlike powers” over their creatures, partly because both the sims themselves, and their environment, are entirely dependent on what the creators do:

In some cases, the relationship [between the sim and its creator] might be literally conceivable as the relationship between deity and creature. Consider an AI in a simulated world, a “Sim”, over which you have godlike powers. … The person running the Sim world might be able to directly adjust an AI’s individual psychological parameters, control its environment in ways that seem miraculous to those inside the Sim (introducing disasters, resurrecting dead AIs, etc.), have influence anywhere in Sim space, change the past by going back to a save point, and more ... Given this relationship, we believe that the manager of the Sim would also possess the obligations of a god (p. 21).

On such a vision, the sims would have few ways of obtaining what they require for a flourishing life (indeed, any life at all), other than through the will of their creators (or whoever gains access to their software and hardware, presumably few in number). The creators would also be free to withhold or supply such resources with little sanction. Both Sandberg and Schwitzgebel & Garza thus express the potential radical vulnerability of the sims on whoever happens to be in charge of their lives and their environs. Since sims are more vulnerable than most other adult human beings, their status as such requires special protection.^4^

Vulnerability on something approaching this scale is widely recognized as raising some ethical worries in the philosophical literature on childhood (Gheaus (2018); Hannan (2018)). However, while it’s a matter of debate whether this kind of vulnerability is good for children, either intrinsically or instrumentally (Skelton, 2018), we can, I think, concede that an indefinitely prolonged period of such vulnerability would be bad for adults, other things equal. Consequently, it looks like creating humanlike sims by using WBE would place them in a condition that is reasonably taken to be bad for them, especially if the original brain is that of an adult’s, and if the sims inherit the moral status of the supplier of the brain they were based on.

One may object to these statements on the disvalue of vulnerability by noting that adult human beings enter many relationships where they make themselves vulnerable—romantic bonds, friendships, bonds of trust, etc.—relationships which give life meaning and are of utmost value. How can one reconcile this undeniable fact with the idea that vulnerability is bad for adults?

Two relevant differences are worth pointing out here: first, while children are dependent for their very survival on their caretakers (and so are the sims), the same *generally* does not apply in romance and friendship. One generally does not need one’s friends and loved ones in order to stay alive. The variation in the degree of vulnerability marks a relevant moral difference between the two cases.

The second difference is that, in most instances, becoming vulnerable to others is a voluntary, uncoerced choice that adults themselves make. The vulnerability is, so to speak, self-imposed. Indeed, even in cases where an adult’s very survival depends on another, say when traveling by plane, the passengers *choose* to become vulnerable in this way. The dependence is unobjectionable partly because it is consensual.

On the other hand, building a sim involves *nonconsensual* imposition of vulnerability—prior to the sim’s creation, of course, it is not able to either accept or reject being created. Consequently, we cannot readily compare the production of sims through WBE to someone choosing to form a bond of friendship or to travel by plane—for the simple reason that the latter two are voluntary (and, ideally, autonomous) choices, wherein the vulnerability is freely accepted as a price worth paying for the promotion of other important interests. Secondly, aside from being freely chosen, these sorts of vulnerability tend not to match the magnitude of what Sandberg (and Schwitzgebel & Garza) describe in the quotations above. *Those* sims are vulnerable to a larger degree, for a much longer time, than most friends and frequent fliers.

It is thus more apposite to compare the emulations’ potential level of vulnerability to that of very small children, except for the fact that the vulnerability may well last for the sim’s entire existence, even as the sim enjoys the mental powers and abilities of a typical human adult. Is it morally acceptable to create such sims, then?

Before proceeding, I will briefly explain what methodology I rely on in answering this question. I take the topic of the ethics of WBE to fall broadly within the umbrella of applied (or practical) ethics. Consequently, I adopt a widely-used^5^ method of pursuing questions in applied ethics that relies on analogical reasoning from intuitively clear cases to reach conclusions about the more difficult cases under discussion. Michael Huemer (2010) describes the method thus:

In my view, most general theories or theoretical approaches in political philosophy—liberal egalitarianism, contractarianism, utilitarianism, and so on—are too controversial to form a secure basis for reasoning. It is not known which, if any, of those theories are correct. I have therefore sought to minimize the reliance on such theories. This does not mean that I assume that all such broad theories are false; I merely refrain from resting my arguments on them. Thus, I do not assume utilitarianism, contractarianism, libertarian rights theory, liberal egalitarianism, nor any general account of harm or rights. Nor do I assume the negation of any of those theories. Instead, *I aim to rest conclusions on widely shared ethical intuitions about relatively specific cases. The method is to describe a case in which nearly everyone will share a particular, clear intuitive evaluation of some action, and then to draw a parallel from the case described to some controversial case of interest*. This methodology follows a well-established tradition in applied ethics. (p. 429, emphasis added)

The subsequent sections adopt just this methodology—I purport to show, through a series of cases eliciting a variety of moral judgments, that whether the vulnerability that Sandberg and Garza & Schwitzgebel describe constitutes an obstacle to creating WBEs depends on what we can expect others to do to the vulnerable; specifically, whether we can expect vulnerability to be exploited. Consequently, what matters for the permissibility of building WBEs, be they emulations of human or non-human brains, depends on what sorts of constraints on others’ behavior there are.

## Vulnerability for Humanlike Sims

Let us now put this methodology to use.

### The Ideal Case

Suppose there is a device that could be implanted, undetected, somewhere in a person’s body, and that can manipulate their brain chemistry in a way that may completely alter their personality, intelligence, moods, emotions and so on.^6^ The device also has a “kill-switch” that, once pressed, instantly kills the victim. The device is operated remotely, and thus can be under the complete control of someone other than the person in whom it’s lodged. It’s indestructible and faultless. With the device in mind (though hopefully not in the brain), consider the following series of cases.

GENIE 1: In Balthasar’s world, there happen to exist a large number of frozen human embryos who, in ordinary course of events, are unlikely to ever develop into adult human beings. One day, a powerful genie visits Balthasar and makes him an offer. One of the embryos, chosen by Balthasar, will, through the genie’s magic, develop into a human child with a high chance at a decent human life. However, at the same time, the genie will implant in the embryo the device mentioned earlier—that Balthasar alone will get to control. After he makes the choice, the genie will disappear, and there will be no further conditions imposed on what Balthasar can do with the knowledge and the power he will have acquired. If Balthasar refuses, the genie will disappear forever, but so will the chance to give the embryo a human life. Suppose, finally, that in Balthasar’s world, every person, including Balthasar, can always be relied on to do what justice requires, and will never prioritize their own wellbeing over doing what’s right.

Is it permissible *for Balthasar* to agree to this offer? Is it permissible *for the genie* to make such an offer in the first place? The question about the genie is, as one may put, *institutional—*it asks whether it is permissible to set up a system of rules that would enable a Balthasar to have this much power over another being*.* The question about Balthasar, in contrast, concerns individual morality—is it permissible *for him* to accept the burden of such power?

Since in our case the genie *knows* that Balthasar won’t abuse his power, it seems clearly permissible for him to make the offer. This is because there is *no risk* of the vulnerability being exploited. Balthasar will always prioritize justice to the child over his own interests, and so will everyone else. Moreover, since in this world no one has to be especially incentivized to pursue justice, we would not need any institutions established to harness people’s motives towards the protection of the vulnerable. They will all do it as a matter of course. Consequently, the genie could make an offer without setting up any system for vetting Balthasar to make sure he’s the right person to carry this burden, or monitoring Balthasar’s subsequent actions to ensure the child’s vulnerability is not in fact abused.

Similarly, since Balthasar himself will do what’s right as a matter of course, including not abusing the arbitrary power over another being, it is permissible for him to agree to the genie’s offer.

### Less than Ideal

Compare the above to a different world:

GENIE 2: As in GENIE 1, except in this world, people are thoroughly unjust; they never or almost never look out for others’ welfare, they exclusively pursue their own selfish ends, and frequently take pleasure in others’ suffering. Often, inflicting such suffering is their most important motivation.

In this hellish world, Balthasar’s decision to agree to the genie’s offer would clearly be immoral, because of the near certainty of the child’s vulnerability being abused. Similarly, it would be wrong for the genie to make this offer in the first place, in virtue of what Balthasar may be expected to do.

Moreover, given the kinds of people populating this world, setting up institutions to temper their depravity would not help either. People would reliably abuse their power and trample over others to achieve their own ends, whether in the role of Balthasar or someone with the power to monitor his actions vis-a-vis the child, or punish him for transgressions.

Unsurprisingly, in the real world, filled with morally imperfect but not depraved humans (for simplicity, assume that in the real world, most people are primarily self-interested, some are like those from the ideal world, and some are from the hellish world), it is far less clear whether it’s permissible for the genie to make the offer. After all, the child would have to remain extremely vulnerable, for the rest of their life (including their adult life), to a person (viz. Balthasar) who *cannot* be counted on to always comply with what justice demands. Rather, while he wouldn’t go out of the way to hurt the child, Balthasar could use his newly-acquired power for his selfish ends, showing indifference for the child’s wellbeing—especially when the two conflict. Balthasar may also encounter knowledge problems: he may simply be too ignorant to help effectively.

Overall, there will be motivational and epistemic constraints on what Balthasar will be able to accomplish. Thus, generally, unless the genie ensures that Balthasar can be incentivized, through an appeal to his self-interest, to effectively protect the child’s wellbeing, Balthasar cannot be reliably counted on to promote it.

I take Balthasar to be analogous, in the relevant respects, to scientists creating WBEs. They too would face the choice whether to bring into existence a being forever consigned to extreme vulnerability. Consequently, it would be permissible for them to create WBEs in the ideal world, and impermissible to do so in the hellish world; what to do in the real world remains unclear. However, some lessons can be drawn about it too: first, it seems like whether it’s morally permissible to impose vulnerability on another person depends, at least in large part, on what others can be expected to do with the power over the vulnerable. The more they can be relied on not to abuse it, the closer we get to the ideal world, and the more justified it becomes to build WBEs.

Secondly, real-world constraints demand that whatever institutions we build to protect the sims are to be designed for the imperfect, flesh-and-blood human beings, not angels or demons. But, crucially, these constraints have to apply not just to the scientists building the sims. We must keep in mind that other actors in the institutional structure: the rule-makers, the enforcers etc., also *cannot* be counted on to always prioritize justice over their own self-interest, come what may. Rather, they’re the kind of people who could use the power they’d acquire for selfish ends, with indifference for others’ wellbeing. They, too, may encounter knowledge problems; they, too, may simply be too ignorant to help effectively.

Thus, institutions have to be structured so that protecting the sims’ wellbeing is in the scientists’ and in others’ self-interest. How do we go about doing this in the real world?

Schematically, it looks like we will need to navigate between two opposite poles: at one extreme, one may think that, *even in the real world*, there are to be no legitimate restrictions on the scientists building WBEs. At the other extreme, one may think that, *even in the real world*, sim production ought to be outlawed. Neither seems particularly attractive; embracing the former would relatively easily allow morally corrupt individuals to achieve the unchecked position of dominance over others and enable them to engage in serious wrongdoing. Embracing the latter would foreclose access to any potentially beneficial effects of sim production (and could facilitate black markets run by unscrupulous individuals). Consequently, the institutional framework governing the creation of WBEs should try to navigate between complete permissiveness and complete restrictiveness.

There are a few potential ways to do so: first, as suggested earlier, we might want a means to *properly vet* the kind of people granted permission to make others vulnerable (something like a license to create WBEs), so that those obviously unfit are not given the option. Secondly, we would want to establish some form of *monitoring* to make sure the vulnerabilities are not in fact exploited (no vetting process will be 100% effective). Thirdly, there ought to be a way of *punishing* those who end up exploiting the vulnerable anyway (no monitoring and prevention will be 100% effective).

This is easier said than done in the non-ideal world. We must design the institutional framework governing the protections of the sims’ interest for imperfect humans, not angels, at all positions within the institutional ladder.

Consequently, it is helpful to look at examples of comparable proposals for institutions charged with protecting the interests of the vulnerable.^7^ For instance, the vetting process could essentially be a form of licensing. Since the sim creators are like parents, in that they too would bring new sentient persons into the world, perhaps it could be akin to *parental* licensing proposed by some philosophers (see e.g. LaFollette (1980)). Unfortunately, such a system may be open to the same dangers, such as special-interest capture, that the opponents of parental licensing raise against the practice. As Christopher Freiman (2022) explains,

even though justified standards of parental competence are available in principle, real-world political forces can work against the application of these standards in practice. … social scientific evidence about parenting is not always the decisive factor shaping parenting regulation. *Politics, rather than the relevant evidence, frequently motivates real-world policy making.* (p. 121, emphasis added)

We can expect similar, perverse incentives to plague WBE licensing. Factors other than the expected quality of sim life can become salient in political decisions about the licenses (especially if money or prestige attach to being able to create sims—which would incentivize the capture of licensing institutions, much as, according to Freiman, the importance of influencing how children are raised would incentivize the capture of parental licensing institutions). Since we are assuming imperfect compliance with justice, whoever ends up with the power to grant such licenses could be tempted to use it to advance their own particular interests, at the expense of the sims’ wellbeing.

In contrast to the parental licensing case, where we are forced to speculate on the real-world outcomes, we do have actual data on the ex-post strategies of monitoring the wellbeing of the vulnerable and punishing the wrongdoers. Again, policies and institutions devoted to protecting children could be our model. Sadly, here too we don’t have reason to be optimistic. A number of empirical studies have found no effect of mandatory reporting and child protective services investigations on the wellbeing of children (see Russell et al. (2018) and references therein), and there is anecdotal evidence of child protection agents simply abusing their position (see Parental Rights Foundation, 2018). This is to be expected, as, first, such services wield considerable power, and, second, they are staffed by imperfect human beings who will sometimes prioritize their own interests over children’s wellbeing, and will sometimes lack the requisite knowledge to make just decisions.

Be it due to incentive or knowledge problems, a similar system of sim protection could produce similarly underwhelming, or even perverse, results (especially if we consider that due to biological and sociological factors, children tend to have a special bond with their parents, and enjoy a special status in society at large; similar bonds are unlikely to obtain between WBEs and their creators, so the internal constraints on abusing them would be comparatively weaker).

Rather than modeling how the sims’ welfare is to be secured on the institutions tasked with protecting children, one could instead place research on sims within the scope of Institutional Review Boards’ (IRBs) jurisdiction, similar to much of the existing human and animal research. Indeed, John Basl suggests that *the lack* of such institutional protections could lead to mistreatment of morally significant artificial entities. Basl says: “Artificial consciousness research, unlike research involving non-human research subjects, is not subject to oversight designed to protect research subjects. *Without oversight* and researcher education, *researchers are less likely to take the welfare of research subjects into account*” (Basl, 2013, p. 28, emphasis added).

Still, such a suggestion remains problematic in the face of empirical work on the effectiveness of IRBs in protecting the interests of study subjects and participants. Some are very critical of IRBs’ record on meeting their stated aims (Zywicki, 2007), while other work emphasizes the lack of workable criteria to assess how well IRBs actually work (Nicholls et al., 2015; Resnik, 2015; Tsan, 2018). Consequently, even if one dismisses Zywicki’s criticisms of IRBs, there seems to be little solid evidence that their introduction into artificial consciousness research would effectively protect the interests of sims.

Moreover, it does not look like institutional protections for sims would help with the problem of vulnerability. Suppose that some ethical mandates are in fact imposed on the researchers on WBE (or artificial consciousness in general). For such mandates to be actually effective, the monitoring and enforcement of compliance would have to be reliable. As I repeatedly emphasized, there is no guarantee of this in the imperfect world.

The same considerations apply to Sandberg’s own brief proposal to safeguard sim security. Sandberg suggests that.

the ethical way of handling brain emulations would be to require strict privacy protection of the emulations and that the emulated persons had legal protection or ownership of the hardware on which they are running, since it is in a sense their physical bodies. Some technological solutions such as encrypted simulation or tamper-resistant special purpose hardware might help. (Sandberg, 2014, p. 452)

The idea seems to be that, in the real world, we can’t trust ordinary people with unfettered power over the sims (for fear of abuse). Consequently, we need legal and technological protections for WBEs.

However, those legal and technological protections would have to be instituted and enforced by the same (kinds of) ordinary people, who, we just assumed, can’t simply be trusted not to abuse their power. Hence, we cannot expect any single person or organization to prioritize the wellbeing of the sims over their own interests (if we could, why not assume that the *creators* of the sims will be so motivated?). So, we need an institutional arrangement able to constrain morally imperfect people.

More generally, and to adopt the conceptual apparatus of the (neo-)republican tradition in political philosophy (see e.g. Pettit, 1997), our conundrum is this: while we need to design systems of rules to protect the sims from subjection to arbitrary power^8^ of their creators (since, as Sandberg and Schwitzgebel & Garza worry, they would be so subjected in the absence of institutional protections), we also need a way to ensure some protection from the arbitrary will of the rule-makers and rule-enforcers. The republican solution to this problem is as follows:

properly-designed democratic institutions should give citizens the effective opportunity to contest the decisions of their representatives. This possibility of contestation will make government agents wielding discretionary authority answerable to a public understanding of the goals or ends they are meant to serve and the means they are permitted to employ. In this way, discretionary power can be subject to popular control in the sense required for a secure enjoyment of republican liberty [from the arbitrary will of another]. (Lovett, 2022, np.)

Applied to sims, the idea would be to ensure that policies aiming to protect and promote their welfare be contestable by citizens, in case they are found objectionable. While a thorough discussion of this way of escaping sim vulnerability is beyond the scope of this paper, one could worry whether, in the non-ideal world, actual voters would care enough about the sims to investigate (difficult) and contest (time-consuming) the laws and regulations pertaining to their wellbeing. Real-world voter behavior does not suggest this is likely to be the case, as most voters are (rationally) ignorant of even the most basic matters of policy and politics (see e.g. Somin, 2016); it’s to be expected they’d also be ignorant of how well the government actually protects the sims, and, hence, incapable of contesting policies they deem inadequate. It’s also questionable whether they would care about the sims enough to examine the real-world effectiveness of sim-protecting policies.

In any case, while not discounting the plausibility of a republican answer to sim vulnerability, below I will sketch an institutional alternative that has the potential to successfully harness individuals’ selfish motivations in the service of sim wellbeing.

### Sim Protection Agencies

In the above-described scenarios, sims lack meaningful exit options. There is usually a monopolistic institution charged with protecting their interests that cannot be escaped if it proves lacking (it’s a further question whether democratic-republican institutions can provide an effective backstop). So in order to improve things, we might need to seek to avoid monopoly and provide the sims with easier exit options.

Assume sims can engage in valuable, productive activities, and, hence, that they are capable of earning an income. Presumably, they would also be willing to avoid situations in which they are easily susceptible to harm at the hands of someone else. Consequently, they would value protection from others’ predations, and be willing to spend some of their incomes on purchasing such protection. This would create a profit opportunity for entrepreneurs capable of offering security services. Under ordinary market conditions, competition for the sims’ custom would emerge. This would incentivize not just attempts to provide adequate protection as cheaply as possible, but also stringent monitoring^9^ of the sims’ wellbeing—as any sim whose welfare was not adequately protected would represent a potential customer.

If there were competition among protection service providers, the sims would not be at the mercy of any particular person, group, or organization—they could switch providers if conditions become disagreeable. Since the sims would be willing and able to pay for protection, providing it would be in the financial interest of the protective agencies. The sims would not have to rely on others’ goodwill alone. So, the sims’ welfare could be protected even in the world where moral motivation is lacking, and people can’t be counted on to fully comply with justice.

Another way to avoid reliance on the goodwill of any particular individual or group would be to enable the sims to pick and choose their *citizenship* at will. This would create the opportunity for competition between different jurisdictions to provide as good conditions as possible in exchange for additional tax revenues from the sims (‘no protection without taxation’). If sims were able to “vote” with their virtual “feet,” this could mimic the salutary aspects of market competition between protection agencies sketched above.

To be sure, these are merely schematic solutions, with many details yet to be filled. However, they offer one more blueprint (in addition to the ones modeled on existing and proposed centralized solutions subject to democratic oversight) for how a more equitable life for the sims could be achieved.^10^

## Vulnerability, Institutions, and Animal Sims

Would it be permissible to produce sims whose cognitive capacities are much more limited than an average human being’s, such as, for instance, WBEs of a cat or a dog? How would their vulnerability figure into this question?

Consider an analogy first:

STRAYS: Suppose all stray kittens and puppies in your city have an unfortunate condition: they will die before their nervous systems develop enough to register any stimulus as either desirable or undesirable; that is, nothing in their lives up to death will have been experienced as either beneficial or harmful. Thankfully, a genie shows up and presents you with a solution: before the animals die, the genie will place them as pets with willing families. As it turns out, this is the *only* way to preserve the animals’ lives. Absent the genie’s intervention they will die without having developed their nervous systems any more.

Again, we can analyze this from the perspective of the ideal, the hellish, and the real world. In the ideal world of strict compliance with justice, it seems obviously permissible to agree to the genie’s proposal—and for the genie to make the proposal in the first place.^11^ In the hellish world, where people enjoy others’ suffering, it seems obviously impermissible to do it. In the real world, things are more difficult, and, once again, we should be looking at institutional arrangements that harness people’s selfish motives to protect the animals’ (and, analogously, the animal-like sims’) interests. The more effective such arrangements are, the closer the real world approaches the ideal (not in the sense of people getting morally better, but rather in the sense of them being motivated to do what’s just); the closer it gets to the ideal, the more justified one is in accepting the genie’s offer.

As we saw, enlisting something like IRBs to handle these issues could potentially be problematic, and the effectiveness of such a system may, at best, be hard to assess. What about other existing templates? Perhaps the most obvious one would be to extend the protections of the animal welfare legislation, such as the Animal Welfare Act (AWA) in the US, to cover non-human sims.

This, too, is easier said than done. For starters, the AWA infamously excludes farm animals, as well as rats and mice (the two species used most frequently in research) from its purview (The Humane League, 2021), depriving them of the legal protection afforded to, say, pets. Who’s to say (some) animal-like sims wouldn’t face the same fate? Secondly, according to some scholars, the actual provisions of the law are underenforced (Swanson, 2001; Vandreau, 2005), leading to more animal suffering than before the law permits on paper. Lastly, in view of others, AWA is actually detrimental to animal welfare (Marceau, 2018).

Consequently, it is questionable whether similar laws and institutions could help with protecting non-human sims, in light of the fact that they seem not to protect many of the most abused, most vulnerable non-human animals. Given human moral frailty, we have no guarantee that they would serve the interests of the sims, rather than some other, better-organized interest groups.^12^

Worryingly, the analog of the competitive solution suggested earlier will likely not work with animal-like sims. This is because it would be inapplicable to entities whose own choices are more difficult (or even impossible) to communicate,^13^ and who would likely be legally unable to enter into contracts and other formal agreements. *A fortiori*, they would be unable to contract for protective services or apply for citizenship. Hence, their prospects for defending themselves or exiting abusive relationships would be seriously diminished.

But maybe we needn’t despair. A different solution could be to allow others with legal standing to *sue* on behalf of the abused sims (and to allow them, were the suit successful, to keep some of the awarded damages to themselves). Threats of lawsuits could disincentivize WBE creators from abusing their sims, even if the sims themselves lack the ability to voice their grievances and to exit the relationship. On the other hand, it would also provide incentives for monitoring the welfare of the sims themselves—finding an abused sim would mean a potential profit opportunity for some entrepreneurial spirit or compassionate soul. Of course, this could also generate something of a perverse incentive (it would be in prospective plaintiffs’ financial interest for the sims to actually be abused), but one can imagine the development of additional institutions (maybe companies selling liability insurance to the people who build sims) whose role would be to limit such temptations and protect the sim creators from facing these sorts of threats.^14^

## Conclusion

There is a risk that sims, if produced by WBE, will be highly vulnerable—their wellbeing, and their very survival, will be very much dependent on others, to a greater extent than ordinary humans’. Does this matter morally? I argued that, in the real world, extreme vulnerability does seem to necessitate the development of an institutional framework for protecting the interests of the extremely vulnerable. In light of research on already existing frameworks of this kind, it remains an open question what sorts of institutional protections ought to be pursued to safeguard the sims’ wellbeing, but we should be more optimistic about decentralized, competitive solutions, than centralized, monopolistic ones.

## Footnotes

1 There are also even more general anti-natalist arguments that would find most types of procreation impermissible (see e.g. Benatar (2006)). I will set this general anti-natalism aside, however, and limit my discussion to the specific issue of creating *artificial* minds.

2 Some dependencies can alleviate vulnerability: e.g. in cases where C, D etc. are antagonistic, i.e., the more *unlikely* C is to provide A with x, the more *likely* D is to do so.

3 This conception is distinct from what Vrousalis calls “absolute vulnerability” which basically means susceptibility to harm, but does not require the contribution of other agents.

4 See also Schroeder and Gefenas (2009) for a further development of this aspect of vulnerability.

5 For examples of this methodology in use in a variety of issues in applied ethics, see Thomson (1971) on abortion, Singer (1972) on poverty relief, Flanigan (2014) on compulsory vaccinations, Sparrow (1999) on terraforming, and the already mentioned Metzinger. For more on the epistemic role of intuition in ethics and other areas of philosophy, see e.g., Huemer (2005) and Chudnoff (2013).

6 Imagine, for instance, that, at a press of a button, the person can go from cheerful to depressed, highly intelligent to below-average, calm to irritated and so on.

7 The aim is not to offer an exhaustive critique of all forms of such proposals, but rather to point to issues that, I think, are emblematic of a more systemic concern with monopolistic, centralized real-world solutions—i.e. that they have to rely on an agent or group being able to transcend their self-interest to regulate and enforce things in a just manner.

8 See also Schwitzgebel and Garza (2020), for a discussion of concerns about exploitation of artificial beings like the sims by designing them to be reliable servants of human beings. See Petersen (2011) for a different approach to the same question.

9 Couldn’t this be simply avoided by carrying things out in secret so that no one would ever know sims were being developed? Perhaps—but, first, secrecy would also be an obstacle in any other system; second, presumably, creating WBEs would require substantial amounts of resources and staff—the more people involved, the more difficult to keep things secret; third, protection agencies would be incentivized to find inventive ways (energy use monitoring, perhaps?) of discovering clandestine WBE labs.

10 This system would work if WBEs were entirely new persons. If the WBE procedure preserved personal identity, things would be easier: people to-be-emulated would contract with appropriate agencies for the protection of their emulated lives before they undergo the process.

11 What if you thought that owning animals, say as pets, is, in general, impermissible? In an ideal world, this wouldn’t be a problem, as the potential “owners” would do everything in their power to let their non-human sims roam as free as allowed by their hardware and programing. I remain silent on whether it’s permissible to own animals as pets in the real world. For a discussion of these ideas see, e.g., Francione and Charlton (2016) and du Toit (2016). If it turned out to be impermissible, then owning animals would constitute abuse, and real-world remedies suggested here would apply.

12 However, see Magaña (2022) for a survey of various ways of ensuring political representation for animals. I suspect that if any of those proposals Magana considers were to be an effective safeguard against animal abuse, an analogous protection of animal-like sims could work as well.

13 This is also a source of a different difficulty with such sims—it would be exceedingly difficult to determine, with a sufficiently high level of certainty, whether they are genuinely conscious—perhaps even more difficult than in the case of humanlike sims.

14 As above, this is a schematic sketch of potential solutions that seem to me to be worth exploring, though problems remain: what if we had a”race to the bottom” among jurisdictions who would limit what counts as abuse in order to attract more sim research, for example? I’m not sure how one could solve this problem.
